# Les aspects des frottis cervico-vaginaux chez les femmes vivants avec le VIH suivies à Thiès/Sénégal et association avec le degré d'immunodépression

**DOI:** 10.11604/pamj.2015.22.62.7264

**Published:** 2015-09-23

**Authors:** Mariama Bammo, Pauline Dioussé, Marietou Thiam, Madoky Maguatte Diop, Adama Berthe, Flugence Abdou Faye, Thierno Abdoul Aziz Diallo, Fatou Seck Sarr, Haby Dione, Papa Souleymane Toure, Bernard Marcel Diop, Mamadou Mortalla Ka

**Affiliations:** 1 Centre Hospitalier Régional de Thiès, Sénégal; 2 EPS Mbour, Thiès, Sénégal; 3 EPS Tivaouane, Thiès, Sénégal

**Keywords:** Frottis cervico-vaginal, VIH, Thiès, Sénégal, Pap smear, HIV, Thiès, Senegal

## Abstract

De nombreuses études ont démontré que les femmes infectées par le VIH ont un risque accru de survenue de néoplasies cervicales intra épithéliales. L'association entre les deux affections étant bidirectionnelle, l'objectif était de décrire les anomalies cervicales chez les femmes séropositives au virus de l'immunodéficience humaine (VIH), de rechercher des facteurs associés et de proposer des recommandations en termes de suivi de ces femmes. Il s'agissait d'une étude transversale, multicentrique recensant l'ensemble des frottis cervico-vaginaux (FCV) et des colposcopies des patientes infectées par le VIH entre 2012 et 2014 dans les services de dermatologie de Thiès et de Mbour. Les données étaient recueillies et analysées par le logiciel EPI Info 2012 version 3.5.4. Les tests statistiques ont été effectués avec un seuil de significativité p <0,05. Etaient inclus 125 patientes. L’âge moyen était de 38,98 ± 10.2 ans [20-77]. Il n'y avait aucun signe d'appels dans 82.4%. Le FCV était normal dans 32.8%, inflammatoire dans 44.8%. Les anomalies cytologiques concernaient 22,4% dont, ASC-H (suspicion de lésions de haut grade: 2.4%), LSIL (lésions de bas grade: 8.8%), HSIL (lésions de haut grade: 4%). Leur majorité (60.7%) avaient un taux de CD4 < 500 et étaient au stade 3 de l'OMS dans 64.3%; la biopsie montrait une dysplasie sévère chez 37.5% des patientes ayant pu réaliser cet examen. Deux patientes ont bénéficié d'un traitement curatif notamment l'exérèse chirurgicale. La survenue de dysplasies cervicales même précoces semble être associée à un stade avancé de l'infection VIH. Un dépistage et un traitement précoces sont absolument nécessaires.

## Introduction

De nombreuses études ont démontré que les femmes infectées par le virus de l'immunodéficience humaine (VIH) ont un risque accru de survenue de néoplasies cervicales intra épithéliales [1]. En effet, il existe un taux plus important de persistance des infections à HPV chez les patientes vivant avec le VIH (PvVIH). Cette persistance est associée au développement de lésions dysplasiques. Elle rend compted'une incidence supérieure des néoplasies intra-épithéliales cervicales (CIN) chez les PvVIH. D'autre part la présence de lésions intraépithéliales bien vascularisées et fragiles peut favoriser transmission de l'infection à VIH ou une réinfection avec de nouvelles souches VIH [2–4]. L'association entre les deux affections (VIH et Néoplasies cervicales) étant bidirectionnelle, l'objectif était de décrire les anomalies cervicales chez les femmes séropositives au VIH, de rechercher des facteurs associés et de proposer des recommandations en termes de suivi de ces femmes.

## Méthodes

Il s'agissait d'une étude transversale multicentrique réalisée dans l'unité de traitement ambulatoire (UTA) du centre hospitalier régional de Thiès (situé à 70km de Dakar) et de l'EPS de Mbour (83km de Dakar et 57 km de Thiès) du 1er janvier 2012 au 31 décembre 2014.

Critères d'inclusion: Toutes femmes infectées par le VIH, suivies dans ces deux structures et ayant bénéficié d'un frottis cervico-vaginal.

Critères non inclusion: Patientes non infectées par le VIH

Les patientes étaient classées selon le stade clinique de l'infection et de la maladie à VIH chez l'adulte et chez l'adolescent de l'OMS(2006). Les frottis cervico- vaginaux et les colposcopies ont été effectués par un des gynéco-obstétriciens des deux centres. Les résultats de la colposcopie étaient classés comme suit: col d'aspect normal; présence d'une transformation atypique de grade I (TAGI); présence d'une transformation atypique de grade II (TAG II); présence de signes de gravité (saignements, anomalies de la vascularisation…); colposcopie non satisfaisante [5]. Les frottis ont été traités et lus selon les procédures standards par un des anatomo-pathologistes du laboratoire de l'Hôpital Aristide Le Dantec de Dakar. Les résultats du frottis cervico-vaginal étaient rendus selon la classification de Bethesda [5]. Les colposcopies étaient demandées chez les patientes présentant des anomalies cytologiques suivies d'une étude anatomo-pathologique des pièces biopsiques dont les résultats étaient présentés selon la classification CIN (Cervical Intraepithelial Neoplasia) [5]. Le traitement concernait uniquement la prise en charge des anomalies cytologiques. Les données ont été recueillies sur un formulaire informatisé. Elles comprenaient les données socio démographiques, cliniques, biologiques, thérapeutiques et modalités évolutives. Ces données étaient analysées par le logiciel EPI Info version 3.5.4. (juillet 2012) du CDC d'Atlanta(USA). Les tests statistiques de Khi-carré étaient utilisés pour comparer les variables qualitatives et le test de Student pour celles quantitatives. Le seuil de significativité a été arrêté à p <0,05.

## Résultats

### Etude descriptive

**Figure 1:**
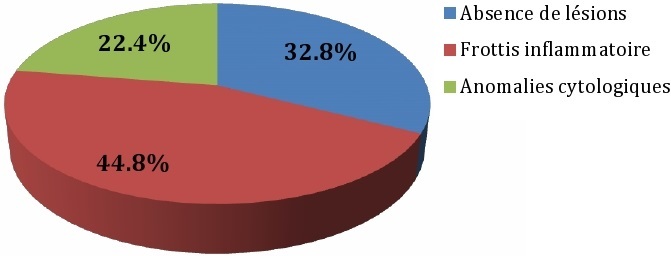
Répartition des femmes selon les résultats des frottis cervico-vaginaux

Sur les 125 patientes incluses dans cette étude 92 (73,6%) étaient issues du Centre Hospitalier Régional (CHR) de Thiès et 33 (26,4%) de l'Etablissement Publique de Santé (EPS)de Mbour, soit une prévalence respective de 0,18 et 0,05. L’âge moyen était de 38,98 ans ± 10.2 ans avec comme valeurs extrêmes 20 et 77 ans. Les catégories socioprofessionnelles se répartissent comme suit: 76 ménagères (60,8%), 15 vendeuses (12%), 10 commerçantes (8%), 6 couturières (4,8%), 6 enseignantes (4,8%), 3 coiffeuses (2,4%), 2 secrétaires, 2 serveuses, 2 étudiantes, 1 infirmière, 1 technicienne de surface, 1 teinturière. Selon le statut matrimonial, 64 étaient mariées(51,2%), suivie de 30 veuves (24%), de 27 divorcées (21,6%) et de 4 célibataires. Le délai moyen de consultation après le dépistage du VIH était de 1,09 ± 11,52 mois. Par rapport aux signes d'appels gynécologiques: des métrorragies et une douleur pelvienne étaient retrouvées chez 1 patiente pour chaque cas (Tableau 1). Selon le stade clinique de l'infection et de la maladie à VIH de l'OMS, le stade I était noté chez 33 patientes (26,4%), le stade II chez 19 patientes (15,2%), le stade III chez 61 patientes (48,8%) et le stade IV chez 12 patientes (9,6%). Le VIH 1 concernait 114 patientes soit 91,2% des cas, le VIH2 8 patientes (6,4%) et le double profil 3 patientes (2,4%). Au total 60 patientes avaient un taux de CD4> 500 soit 48% des cas et 65 patientes avaient un taux ≤ 500 (52%). Concernant le traitement anti rétroviral (ARV) nous avons noté 110 patientes sous ARV (88%) et 15 sans ARV (12%) (Figure 1), (Tableau 2). Une colposcopie était réalisée chez 8 patientes (6.4%) avec 2 cas de TAG I (25%), 3 cas de TAG II (37,5%), et 3 cas de signes de gravité (37,5%). L’étude anatomo-pathologiques retrouvait 1 cas respectivement de condylome plan associé à une métaplasie malpighienne, de CIN2 (dysplasie moyenne), et de Carcinome in situ (12,5%); 2 cas de CIN 1(dysplasie légère) soit 25% et 3 cas de CIN 3 (dysplasie sévère) soit 37,5% des cas. Deux patientes avaient bénéficié d'une hystérectomie radicale: 1 pour CIN 3 et l'autre pour carcinome in situ.

**Tableau 1:** Répartition des patientes selon le stade clinique

Stade clinique	Nombre	Pourcentage
Stade I	33	26,4%
Stade II	19	15,2%
Stade III	61	48,8%
Stade iv	12	9,6%
Total	125	100%

**Tableau 2:** Répartition des femmes selon les anomalies cytologiques retrouvées

Anomalies cytologiques	Proportion	Pourcentage (%)
ASC-US(indéterminé)	9	10.7%
ASC-H (suspect)	3	32.1%
LSIL (bas grade)	11	39.3%
HSIL (haut grade)	5	17.9%
Total	28	100%

### Facteurs associés aux anomalies cytologiques

La durée moyenne de traitement ARV chez ces patientes était de 4 ans (10 mois -7 ans) (Tableau 3). Concernant le profil sérologique: 24 patientes étaient VIH 1 (85,7%), 3 étaient VIH 2 (10,7%) et 1 VIH 1 + 2: 1 (3,6%) (Tableau 4).

**Tableau 3:** Répartition des anomalies cytologiques selon le stade OMS

Stade OMS Anomalies cytologiques	Stade 1	Stade 2	Stade 3	Stade 4
ASC-US (indéterminé)	3	1	5	0
ASC-H (suspect)	0	1	1	0
LSIL (bas grade)	1	1	8	2
HSIL (haut grade)	1	0	4	0
Total	5	3	18	2

**Tableau 4:** Répartition des anomalies cytologiques selon le taux de CD4

Taux de CD4 Anomalies Cytologiques	CD4 ≤ 500	CD4 >500
ASC-US(indéterminé)	3	6
ASC-H (suspect)	2	1
LSIL (bas grade)	8	3
HSIL (haut grade)	4	1
Total	17	11

Etude analytique: aucun lien statistiquement significatif n’était retrouvé.

## Discussion

Sur les 125 FCV réalisés dans notre série, 28 présentaient des anomalies cytologiques (22,4%). Nos résultats se rapprochent de ceux de Koffi et al [6] mais sont supérieurs à ceux de N'guessan E. et al. qui a retrouvé 18 cas soit 11,76% [2]. L’âge moyen retrouvé dans notre série est similaire à celui retrouvé dans l’étude de N'guessan E. et al. [2]. Ceci pourrait s'expliquer par le fort taux de séroprévalence chez les femmes au Sénégal qui se situe entre 15 et 49 ans selon l'EDS 5 [7]. Le VIH 1 prédominait dans notre série ce qui épouse le profil épidémiologique de l'infection par le VIH au Sénégal comme dans la plupart des études menées dans l'Afrique de l'Ouest [2]. Le délai moyen de consultation après le dépistage VIH était court en conformité avec la stratégie de suivi et de prise en charge des PVVIH par le CNLS [8]. Les frottis étaient inflammatoires avec quasiment le même pourcentage dans notre série que dans celle de Koffi et al. [6]. Parmi nos patientes présentant des anomalies cytologiques, seules deux avaient des signes d'appels gynécologiques (métrorragies et douleurs pelviennes). En effet, les stades précancéreux du cancer du col de l'utérus sont souvent asymptomatiques; de même, le cancer du col de l'utérus n'entraîne souvent que très peu de troubles, et ce n'est qu’à des stades avancés que des symptômes se manifestent, d'où l'intérêt du dépistage [9]. La majorité des patientes ayant des anomalies cervicales étaient classées au stade 3 de l'OMS (18 patientes) et avaient un bas taux de CD4 (17 patientes) comme dans l’étude de Couture MC et al. au Cambodge [10] et SOBESKY M. et al. en Guyane [11]. NGuessan et al. en Côte d'ivoire ont trouvé un lien statistique [2], contrairement à Fridman qui n'a trouvé aucun lien avec le degré d'immunodépression ou la prise d′un traitement antirétroviral [12]; cette différence serait probablement dû à la diversité des méthodologies et à la taille des échantillons (Tableau 5).

**Tableau 5:** Comparaison des résultats cytologiques de notre série avec celle de Ceccaldi

Anomalies cytologiques	Ceccaldi et al N= 18	Notre série N = 28
ASC-US	4 (7,84%)	9(10,7%)
ASC-H	0	3(32,1%)
LSIL	15(29,41%)	11(39,3%)
HSIL	7(13,73%)	5(17,9%)

**ASC-US**: atypies des cellules malpighiennes de signification indéterminée; **ASC-H**: atypies des cellules malpighiennes ne permettant pas d'exclure une lésion intraépithéliale de haut grade; **LSIL**: lésion malpighienne intraépithéliale de bas grade;**HSIL**: lésion malpighienne intraépithéliale de haut grade.

Nos résultats se rapprochent de ceux de Ceccaldi P. et al. [13] concernant la prédominance des lésions de bas grade parmi les résultats cytologiques. Par rapport à la colposcopie, elle concernait seulement 8 patientes dans notre série contrairement à l’étude de FRIDMANN S. chez qui 100% des patientes ayant des anomalies cervicales auraient bénéficié d'une colposcopie/biopsie systématique ce qui lui a valu 13 CIN 1 et 1 CIN 2; cependant il n'a retrouvé aucun cas de cancer invasif [12]. Par contre, nous avons rapporté dans notre série que la biopsie montrait: 2 CIN 1, 1 CIN 2, 3 CIN3 et un cas de carcinome in-situ. Le faible taux de réalisation des colposcopies pourrait être dû au fait que 60,8% de nos patientes sont de femmes au foyer et ces examens complémentaires étaient à leur frais. Nos résultats sont largement sous-estimés compte tenu de la faible réalisation de cet examen, mais relativement plus alarmant que ceux de FRIDMANN S. qui rapportaient uniquement des dysplasies légères(CIN1) et moyennes(CIN2) [12]. Deux de nos huit patientes ont pu bénéficier d'un traitement curatif. Ce qui montre les limites de notre étude qui dénotent du coût élevé du diagnostic et de la prise en charge de ces patientes dans nos pays en voie de développement.

## Conclusion

Notre étude a montré une fréquence plus élevée, non significative d'anomalies cytologiques même précoces à un stade avancé de l'infection VIH. Toutefois, la surveillance des infections par le HPV et le dépistage des anomalies cytologiques par un frottis cervico-vaginal annuel doivent être obligatoires afin d’éviter l’évolution vers un cancer invasif.
